# Content-Aware Focal Plane Selection and Proposals for Object Tracking on Plenoptic Image Sequences

**DOI:** 10.3390/s19010048

**Published:** 2018-12-22

**Authors:** Dae Hyun Bae, Jae Woo Kim, Jae-Pil Heo

**Affiliations:** 1 Department of Electrical and Computer Engineering, Sungkyunkwan University, 2066 Seobu-ro, Jangan-gu, Suwon-si, Gyeonggi-do 16419, Korea; noahark0628@gmail.com; 2 Electronics and Telecommunications Research Institute, 218 Gajeong-ro, Yuseong-gu, Daejeon 34129, Korea; jae_kim@etri.re.kr; 3 Department of Software, Sungkyunkwan University, 2066 Seobu-ro, Jangan-gu, Suwon-si, Gyeonggi-do 16419, Korea

**Keywords:** plenoptic imaging technique, object tracking, bounding box proposal, content-based image matching

## Abstract

Object tracking is a fundamental problem in computer vision since it is required in many practical applications including video-based surveillance and autonomous vehicles. One of the most challenging scenarios in the problem is when the target object is partially or even fully occluded by other objects. In such cases, most of existing trackers can fail in their task while the object is invisible. Recently, a few techniques have been proposed to tackle the occlusion problem by performing the tracking on plenoptic image sequences. Although they have shown promising results based on the refocusing capability of plenoptic images, there is still room for improvement. In this paper, we propose a novel focus index selection algorithm to identify an optimal focal plane where the tracking should be performed. To determine an optimal focus index, we use a focus measure to find maximally focused plane and a visual similarity to capture the plane where the target object is visible, and its appearance is distinguishably clear. We further use the selected focus index to generate proposals. Since the optimal focus index allows us to estimate the distance between the camera and the target object, we can more accurately guess the scale changes of the object in the image plane. Our proposal algorithm also takes the trajectory of the target object into account. We extensively evaluate our proposed techniques on three plenoptic image sequences by comparing them against the prior tracking methods specialized to the plenoptic image sequences. In experiments, our method provides higher accuracy and robustness over the prior art, and those results confirm that the merits of our proposed algorithms.

## 1. Introduction

Object tracking is one of the most active research topics in computer vision, since it is a core technique in various industrial applications such as video-based surveillance systems and autonomous vehicles. The goal of the object-tracking problem is to estimate the trajectory of target object in a sequence of images. Given an initial bounding box containing the target object at the beginning of the tracking task (i.e., frame index t=1), the object tracker is asked to compute bounding boxes of the target object in subsequent frames (i.e., frame index t>1). Although there are numerous trackers for multiple-object tracking, we restrict our discussion in this paper on the problem of single object tracking since our proposed techniques are orthogonal to such problem configurations.

There are various factors that make object tracking difficult such as the change of appearance of the object due to the rotation, translation, or illumination. Among those factors, one of the most difficult cases is when the target object becomes partially or even fully occluded. The traditional tracking algorithm designed for 2D image sequences are highly likely to be missing the target object while it is being invisible. A few tracking techniques [1,2,3] have used the refocusing capability of plenoptic images to overcome the occlusion problem. Even though the object is occluded, changing the focus to the plane where the target object is maximally focused makes the target object visible while the occluders are being blurred out. Cho and Javidi [1] have reduced the occlusion problem using integral imaging technique to discover the occluded target object based on plane-by-plane reconstruction. Kim et al. [2] have presented a focus index selection method based on a focus measure [4] that identifies the maximally focused focal plane and perform the object tracking on the selected images. A follow-up work [3] has validated that applying image sharpening filters and a simple proposal technique can enhance the accuracy and robustness of [2]. Aforementioned methods have proposed solutions for the occlusion problem by using plenoptic imaging techniques and shown noticeable tracking results; however, none of them completely solved the problem. Specifically, focal plane selection algorithm in [2] can miss the optimal focal plane since their method is only relied on the focus measure, and the proposal technique of [3] does not fully use the advantage of plenoptic images.

In this paper, we propose a novel focal plane selection algorithm that takes both focus measure and visual similarity into account for the object tracking on plenoptic image sequences and design a bounding box proposal method tailored to our selected focus index. The main contributions of this paper are summarized as follows:

- We propose a novel focus index selection algorithm, *content-aware focal plane selection*, that identifies the maximally focused and visually consistent focal plane. While the prior techniques [2,3] have only used the focus measure, our selection method additionally consider whether the target object is clearly appeared in the image plane (Section 4).
- We propose a novel bounding box proposal scheme based on the optimal focus index computed by our content-aware focal plane selection algorithm. Since the focus index is directly related to the distance between the camera and object, we use the change of the focus index to accurately estimate the scale of the object in the image plane. We also design an algorithm to propose the location of candidate bounding boxes by modeling of the trajectory of the object. We generate several candidate bounding boxes based on the both scale and motion factors and select the best one among them according to the visual similarity to the target object (Section 5).

**Figure 1:**
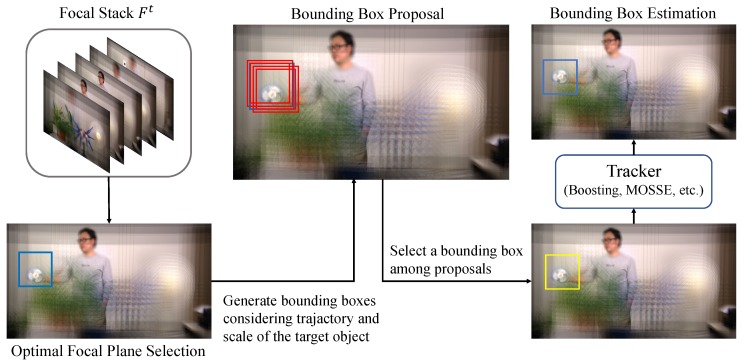
Procedure of our object-tracking techniques for plenoptic image sequences.

Once we identify a focus index and a bounding box proposal based on our proposed techniques, the remaining task is reduced to an ordinary object-tracking problem in 2D images. Since our method does not have any restriction on the choice of trackers, we can use any existing tracking techniques to estimate the location of object. In other words, we can take advantage of advanced tracking techniques. Figure 1 illustrates the process of the object tracking task in plenoptic image sequences. Please note that the plenoptic images can be captured by not only specialized equipment but also micro lens arrays which can be installed to portable commodity devices such as smartphones. Once the plenoptic images are acquired, they can be used in any computing devices including PCs, smartphones, and tablet.

We have extensively evaluated our proposed techniques on three different plenoptic image sequences. To show the flexibility of our method in terms of choice of trackers, we have applied three different tracking engines, Boosting [5], minimum output sum of squared error (MOSSE) [6], and MOSSE with convolutional neural networks (CNN) features [7] trackers to our proposed scheme. Experimental results show that our techniques provide both higher accuracy and robustness over the recent methods [2,3] (Section 6).

## 2. Related Work

Object tracking is one of the fundamental problems in computer vision. Due to its importance, there has been numerous techniques to solve the problem. Excellent surveys for those methods are available [8,9,10].

Among several challenges in the object tracking, one of the most difficult cases is dealing with the target object partially or even fully occluded. There have been many studies that have attempted to resolve such occlusion problem. Adam et al. [11] have proposed a representation of the target object based on multiple image fragments which is robust to the partial occlusions or appearance changes. A tracking method based on a sparse dictionary for modeling the appearance of the object is proposed in [12]. They constructed a confidence map from the sparse representation, and then located the object by voting and mean-shift. Zhang et al. [13] also reconstructed an appearance of object by sparse measurement matrix exploiting a non-adaptive random projection. Grabner et al. [14] employed the visual context into the object tracking task. Even if the target object becomes invisible, they can infer its location based on the motion of correlated objects in the scene. Babenko et al. [15] proposed multiple instance learning to train a classifier to learn an appearance of the object in robustly so that the tracker alleviates the problem of partial occlusion. A dynamic graph-based tracker has been proposed to address both deformation and occlusion problem in [16].

Tracking algorithms based on the correlation filters show remarkable results in recently object tracking challenge [17]. Such trackers learn the appearance of the target object. Bolme et al. [6] have proposed an object tracker based on a MOSSE filter. The MOSSE-based tracking algorithms compute a response map with the correlation score based on a correlation filter and an image patch. The scores can be computed by a simple convolution operation in Fourier Transform domain to achieve high efficiency in terms of computational costs. A new location of the target object is then identified by centering the peak among the correlation scores of the response map. Once the position of the target object is estimated, the correlation filter is incrementally updated with an image patch cropped from the location of the object.

There has been various work proposing improved algorithms based on the correlation filters by exploiting its simplicity and efficiency. Notable examples include a kernelized correlation filters [18], a filter trained from multiple dimensional features instead of a single channel of images [19], spatial regularization to resolve periodic boundary effects [20], and using CNN features to training correlation filters [7]. Lukezic et al. [21] uses spatial constraints to overcome the limitations regarding to the assumption of shape of the target object. Shi et al. [22] have proposed a sampling technique by considering the spatiotemporal consistency for training correlation filters. An adaptive method to adaptively update the kernelized correlation filters according to the scene classification is proposed in [23]. Also, Xue et al. [24] attempted to improve tracking performance by reducing noises from background. They adopt the concept of cellular automata to update an appearance template.

In this paper, our contribution is more focused on handling plenoptic image sequences not concentrating on development of a new tracking algorithm working in 2D images. Our technique is designed to accept any of existing tracking methods. We, however, mostly tested our method by combining with MOSSE tracker and its variations to take advantage of their simplicity and efficiency.

**Figure 2:**
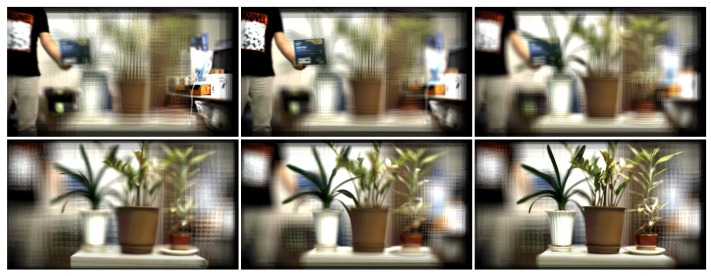
Example of plenoptic images.

On the other hand, the refocusing capability of plenoptic imaging technology inheritably enables to reduce the occlusion problem in the object tracking task. Cho et al. [1] have proposed a tracking method in the integrated image sequences. A plenoptic image sequence is consisting of focal stacks, and each focal stack is corresponding to each frame index and containing a set of images reconstructed in a plane-by-plane manner. Thus, it is crucial to select an appropriate focal plane where the target object is maximally focused. By selecting a proper focus index, the object can become visible even it is blocked by occluders. Figure 2 shows examples of plenoptic images.

Kim et al. [2] shows promising results by using the focus measure [4,25] to select focal planes and performing tracking on those image planes. The technique [2] is extended in Bae et al. [3] that proposed two additional steps. They performed an image sharpening process prior to estimate the location of the object, since the selected images determined by the focus measure still contain blurry reasons due to the discreteness of focal planes. They also presented a heuristic to generate proposals based on a fixed ratio from the previous frame. We found that, both techniques [2,3] can identify an undesired focus index since their focal plane selection is strongly relied on the focus measure. If there is an occluder that can have higher focus measure score than the target object, their algorithms may select the focus index where the occluder is maximally focused instead of the target object. To overcome such problem, we propose a novel focus index selection technique that considers both the focus measure and visual consistency.

Recently deep CNNs have shown state-of-the-art performance in several computer vision tasks including object classification [26,27,28,29,30] and detection [31,32,33]. The network trained for solving image classification problem can produce generic features of images, the pre-trained CNN models can be employed as image feature extractor. For instance, in the object detection problem region proposal-based architectures [31,32] use VGG [27] to extract spatial and semantic information from input images. Inspired from the success, deep features extracted from CNNs are actively used in the object tracking. Wang et al. [34] have proposed an online tracker by learning generic features of natural images based on an autoencoder trained offline. In [35], a CNN that learns relationships between the appearances and motions of objects is proposed to predict the position of a particular object in the next frame. Danelljan et al. [7] have used deep CNN features to train correlation filters instead of using single gray scale image. They specifically used activations of a convolutional layer which are multiple channels data. They have chosen the first activations of convolutional layer as CNN feature to preserve spatial information. The proposed method achieved remarkable results on three public benchmark datasets [8,17,36]. On the other hand, Babenko et al. [37,38] have shown that the features extracted from CNNs are powerful image descriptors in image matching or retrieval tasks. In this paper, we validate our proposed technique that can take advantage of the success of deep learning in various computer vision tasks. We specifically combine our method with the MOSSE tracker trained by CNN features and use the features to select an appropriate focal plane based on the visual consistency. More details are discussed in Section 4 and Section 5.

## 3. Problem Formulation and Overview

### 3.1. Problem Formulation

Let us first define notations that we will use throughout this paper. A plenoptic image sequence I consists of *N* frames of focal stacks, and a focal stack at the frame index *t* is then denoted by $F^{t}$. The focal stack $F^{t}$ of the frame *t* is defined as a set of images, $F^{t}=\left\{I_{1}^{t},I_{2}^{t},...,I_{M}^{t}\right\}$, where *M* is the number of focal planes in the stack. As a result, the plenoptic image sequence I of *N* time frames and *M* focal planes has NM images, $\left\{I_{k}^{t}\right\}$ where t∈{1,2,...N} and k∈{1,2,...,M}.

The object tracking on 2D image sequence is a well-defined problem that estimates a trajectory of an object over the time. Specifically, the estimated locations of the target object at the frame index *t* is generally described by a bounding box $B^{t}$ centered at $\left(x^{t},y^{t}\right)$ whose width and height are $w^{t}$ and $h^{t}$, respectively. Given an initial bounding box $B^{1}$ of the target object, the object tracking is to find a sequence of bounding boxes $B^{2},...,B^{N}$ containing the target object over the frames.

Object-tracking problems on a plenoptic image sequence and a simple 2D image sequence share the same goal locating the target object over frames. However, the problem on a plenoptic sequence is more complex than an ordinary image sequence because it requires additional consideration to identify appropriate focal planes in the focal stacks. In conventional object-tracking problem, it is assumed that the target object is well-focused, and its appearance is fairly clear in 2D images. On the other hand, in the plenoptic case the region of a target object can be blurry or even occluded if we select an inappropriate focal plane. Thus, in each frame *t* we need to first carefully identify an optimal focal plane where the object region is visible and clearly presented prior to estimate the location of the object. The visibility and clarity are directly related to the success-failure rate and accuracy of the tracking result.

### 3.2. Overview

To estimate the trajectory of a target object in the plenoptic image sequence, we first select an optimal focal plane from a focal stack $F^{t}$ at each frame *t*. We propose a novel algorithm to identify an optimal focal plane based on both the focus measure [25] and visual feature similarity (Section 4). Compared to the prior attempts for the optimal image selection based on the focus measure [2], our proposed technique exploiting visual feature similarity provides higher robustness and accuracy. Once an optimal image is identified, we generate several candidate bounding boxes based on two factors, the history of the object trajectory and distance between the object and camera. We then select the best one among the candidates according to the image patch similarity to the tracking result at the previous frame (Section 5). Finally, the selected bounding box is passed to the object-tracking module designed for conventional 2D image sequences to obtain a refined tracking result.

## 4. Focus Index Selection

As discussed in Section 3.1, identifying an optimal focal plane from a focal stack is crucial in the object tracking on plenoptic image sequences since the visibility and clarity of appearance of the target object are highly correlated with the success-failure rate and accuracy of the tracking task. Moreover, a correctly selected focal plane reduces the difficulty in the tracking when the target object is partially or even fully occluded by other environmental objects. In this section, we introduce our novel algorithm to select an optimal image from a given focal stack.

### 4.1. Focus Measure Based Focus Index Selection

To select an optimal image from the focal stack, one reasonable approach is using the focus measure. In this strategy an optimal image is defined to be the maximally focused at the region where the target object is assumed to be located. Specifically, given a region of interest (RoI) defined as a set of pixels Ω(x,y) where the target object highly likely to be located (e.g., the bounding box $B^{t-1}$ estimated in the previous frame t-1), the optimal image $I_{\hat{k}}^{t}$ is selected among a set of images $\left\{I_{1}^{t},...,I_{M}^{t}\right\}$ in the focal stack $F^{t}$ to have the highest focus measure on Ω(x,y). Pertuz et al. [4] have conducted extensive evaluation of many successful focus measures and reported that Sum-Modified-Laplacian (SML) operator [25] provided high performance and was robust to various factors such as the quality and content of the image. The focus measure based on SML operator for an image *I* and RoI Ω(x,y) is defined as follows:

(1) $$\begin{array}{c} \phi \left(I,\Omega \left(x,y\right)\right)=\sum_{\left(i,j)\in \Omega (x,y\right)}\Delta_{m}I\left(i,j\right), \end{array}$$

where $\Delta_{m}I\left(i,j\right)$ is a modified Laplacian with two convolution filters $L_{X}=\left[-1\;2\;-1\right]$ and $L_{Y}=L_{X}^{T}$ as expressed as follows (∗ is the convolution operator):

(2) $$\begin{array}{c} \Delta_{m}I\left(i,j\right)=|I*L_{X}|+|I*L_{Y}|. \end{array}$$

Let us define an image cropping function c(I,B) that extracts an image patch corresponding to a bounding box B(x,y,w,h) from the image *I*. For instance, c(I,(15,30,10,20)) produces a rectangular image patch from *I* whose coordinates of left upper and right lower are (10,20) and (20,40), respectively. We then can define the *focus score*, $f-\mathrm{score}(I_{k}^{t})$, of an image $I_{k}^{t}$ in the focal stack $F^{t}$ by rewriting the focus measure (Equation (1)) by using the cropping function c(·,·) as follows:

(3) $$\begin{array}{c} f-\mathrm{score}\left(I_{k}^{t}\right)=\sum_{\left(i,j\right)\in c\left(I_{k}^{t},B^{t-1}\right)}\Delta_{m}I_{k}^{t}\left(i,j\right) \end{array}$$

The focus index selected according to the focus score can provide a reasonably accurate focal plane as shown in [2,3]. Using only the focus score to select an index, however, can produce an undesired focus index as the optimum in cases where the target object is occluded by environmental objects which is maximally focused. In [2], a hill-climbing technique is proposed to resolve the issue. The heuristic starts from the focus index ${\hat{k}}_{t-1}$ of the previous frame t-1, and searching for the focus index having a local peak of focus score by moving to higher scores. Since the algorithm prevents significant changes in the focus index by starting from the focus index of the previous frame, it could reduce the aforementioned problem. However, it still suffers from such incorrect index selection when there is the occluder close to the target object but has higher focus score than the target.

### 4.2. Content-Based Score for Focus Index Selection

The aforementioned issue discussed in Section 4.1 is mainly because the focus index selection only based on the focus measure (Equation (1)) which is totally unaware of the content in the RoI. When evaluating the optimality of given focus index, it is also crucial factor whether the target object appears or not. We, hence, propose to use visual similarities in the focus index selection to capture the content in the RoI.

We define an image feature extractor f(I) that produces a high-dimensional feature representation describing the content of a given image *I*. Notable examples of such feature extractor f(I) include the Bag of Visual Word (BoVW) [39] and Neural Codes [37].

Given a focal stack $F^{t}$ at the frame index *t*, we evaluate images $I_{k}^{t}$ from the focal stack by examining image patches $c(I_{k}^{t},B^{t-1})$ corresponding to the estimated bounding box $B^{t-1}$ at the previous frame. We define a *content score*, $c-\mathrm{score}(I_{k}^{t})$ of the *k*th image in the focal stack at frame *t* to be the average similarity of $c(I_{k}^{t},B^{t-1})$ to the initial bounding box at the first frame and the estimated bounding box at the previous frame t-1, as follows:

(4) $$c-\mathrm{score}\left(I_{k}^{t}\right)=\frac{1}{2}\{f-\mathrm{sim}(f(c\left(I_{{\hat{k}}_{1}}^{1},B^{1}\right)),f(c\left(I_{k}^{t},B^{t-1}\right)))+f-\mathrm{sim}(f(c\left(I_{{\hat{k}}_{t-1}}^{t-1},B^{t-1}\right)),f(c\left(I_{k}^{t},B^{t-1}\right)),$$

where $f-\mathrm{sim}(f_{x},f_{y})$ defines the similarity of the features $f_{x}$ and $f_{y}$. Please note that our content score can be simply accepting any feature similarity measures including Euclidean distance, cosine similarity, and even more complex metrics such as Mahalanobis distances. This implies that we can take advantage of any advanced image feature representation since our method is orthogonal to the features or their similarity. For instance, our content score becomes the followings if the feature similarity is defined by dot products (i.e., cosine similarity):

(5) $$\begin{array}{ccc} c-\mathrm{score}(I_{k}^{t}) & = & \frac{1}{2}(f(c\left(I_{{\hat{k}}_{1}}^{1},B^{1}\right))\cdot f(c\left(I_{k}^{t},B^{t-1}\right))+f(c\left(I_{{\hat{k}}_{t-1}}^{t-1},B^{t-1}\right))\cdot f(c\left(I_{k}^{t},B^{t-1}\right)))\\ & = & \frac{f(c\left(I_{k}^{t},B^{t-1}\right))}{2}\cdot (f(c\left(I_{{\hat{k}}_{1}}^{1},B^{1}\right))+f(c\left(I_{{\hat{k}}_{t-1}}^{t-1},B^{t-1}\right))), \end{array}$$

In the experiments, we have used VGG-16 [27], SqeezeNet [40] and Inception-ResNet v2 [30] networks to extract features. We crop the image patch and resize to suitable sized image for the network. To capture the semantic similarity among image patches, the features are extracted from the last layer except the classification layer. The extracted features are then normalized according to their $L_{2}$ norms. In this case, we can use Equation (5) to determine the similarity.

### 4.3. Combined Score and Focus Index Selection

Using the content similarity alone to select an optimal image from the focal stack can cause a problem when there are occluders which have similar appearance to the target object. Suppose that the target object which is maximally focused at focus index $k_{target}$ moves to the behind another object which has very similar or even identical appearance to the target object. In this scenario, our content similarity measure can be failed to identify the focus index $k_{target}$ by selecting the focus index where the occluder is maximally focused, if the feature similarity is the highest with the occulder.

To resolve such problem, we finally combine the aforementioned two scores, focus and content scores (Equations (3) and (4)), to examine the optimality of focal planes. Since the values computed by those two scoring methods are in different ranges, we convert them to the relative value with respect to the scores of the selected focus index in the previous frame $I_{{\hat{k}}_{t-1}}^{t-1}$. As a result, our combined score for the focus index, $\mathrm{fi}-\mathrm{score}(I_{k}^{t})$ is defined as follows:

(6) $$\begin{array}{c} \mathrm{fi}-\mathrm{score}\left(I_{k}^{t}\right)=\frac{1}{2}\cdot \left(\frac{f-\mathrm{score}(I_{k}^{t})}{f-\mathrm{score}(I_{{\hat{k}}_{t-1}}^{t-1})}+\frac{c-\mathrm{score}(I_{k}^{t})}{c-\mathrm{score}(I_{{\hat{k}}_{t-1}}^{t-1})}\right). \end{array}$$

**Figure 3:**
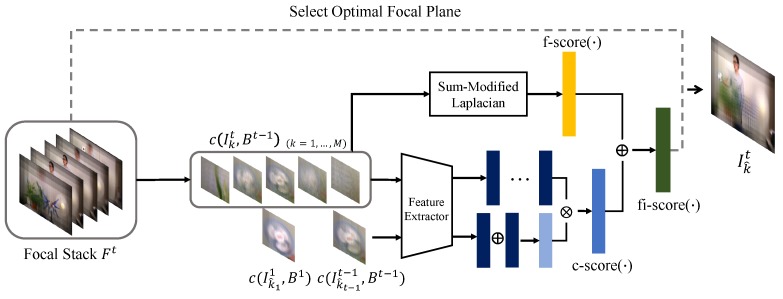
Illustration of our focal plane selection method.

**Figure 4:**
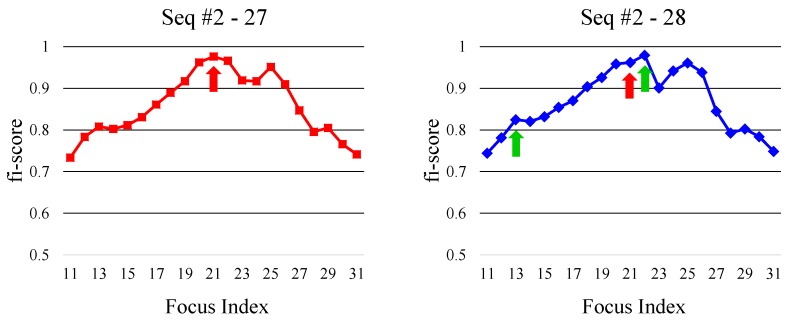
The left and right graphs show the fi-scores with respect to the focus index at 27th and 28th frames of Seq #2 (Section 6), respectively. The red arrowed index is the selected focus index at the 27th frame. In the 28th frame, fi-scores are first computed, and two local peaks are searched from the focus index of the previous frame. The green arrowed indices are the identified two indices. We then select closer index from the focus index of the previous frame among two peaks as an optimal focus index. In this example, the right green arrowed index is selected.

To find an optimal focus index where we perform the object tracking, one straightforward approach is taking the index having maximum fi-score(·). This strategy, however, can cause undesired focus index selection as we discussed in Section 4.1. Moreover, the hill-climbing heuristic proposed in [2] can be missing the optimum. We thus propose to search two peaks in terms of fi-score(·), towards decreasing and increasing focus indices. We also start from the optimal focus index ${\hat{k}}_{t-1}$ of the previous frame, and search two focus indices $k_{L}$ and $k_{R}$ which have locally maximum fi-score. Specifically, we search for two indices $k_{L}$ and $k_{R}$ that satisfy the follows:

(7) $$\begin{array}{c} \mathrm{fi}-\mathrm{score}\left(I_{k_{L}-1}^{t}\right)\leq \mathrm{fi}-\mathrm{score}\left(I_{k_{L}}^{t}\right),\;\mathrm{fi}-\mathrm{score}\left(I_{k_{L}}^{t}\right)\geq \mathrm{fi}-\mathrm{score}\left(I_{k_{L}+1}^{t}\right),\\ \mathrm{fi}-\mathrm{score}\left(I_{k_{R}-1}^{t}\right)\leq \mathrm{fi}-\mathrm{score}\left(I_{k_{R}}^{t}\right),\;\mathrm{fi}-\mathrm{score}\left(I_{k_{R}}^{t}\right)\geq \mathrm{fi}-\mathrm{score}\left(I_{k_{R}+1}^{t}\right), \end{array}$$

where $k_{L}\leq {\hat{k}}_{t-1}$ and $k_{R}\geq {\hat{k}}_{t-1}$. Among the indices $k_{L}$ satisfying the conditions, we select the largest index which is the closest to the ${\hat{k}}_{t-1}$. Similarly, we pick the smallest one for $k_{R}$. We then select one of $K_{L}$ and $K_{R}$ closer to ${\hat{k}}_{t-1}$ as ${\hat{k}}_{t}$. If $K_{L}$ and $K_{R}$ have the same distance from ${\hat{k}}_{t-1}$, then we take one having higher fi-score(·). Figure 3 illustrates the overall process of our focus index selection algorithm. And Figure 4 shows a practical example of the optimal focus index selection. Once fi-score is computed for given focal stack, the peak selection algorithm looks for two local peaks in both left and right directions from the focus index selected in the previous frame. Among two peak indices, the one closer to the focus index at the previous frame is then selected as an optimal focus index of the current frame.

Please note that, extensive comparisons between the f-score and fi-score in both quantitative and qualitative manners are available in Section 6.

## 5. Bounding Box Proposal Based on Focus Index

To overcome the difficulty in the object-tracking task caused by the motion and size changes of the object, many of existing tracking techniques first conduct a bounding box proposal stage. The goal of this proposal stage is that producing a candidate bounding box which is nicely fit to the target object.

In the object tracking on plenoptic image sequences, Bae et al. [3] have presented a simple heuristic for bounding box proposal. They suggest generating proposals based on three fixed scales (3% smaller, the same, and 3% larger) from the selected proposal at the previous frame. They then feed each proposal to the Boosting tracker [5] and select the best one among those boxes based on the tracking confidence score determined by the Boosting tracker. Although this heuristic has been reported to provide accuracy enhancement over the baseline, using fixed scaling ratio is still too ad-hoc and their method is highly depending on the Boosting tracker. In other words, it is hard to take advantage of other advanced trackers. One may think of alternative proposal methods that use object detectors. In such approaches, the proposal is selected among the detected object regions according to the similarity to the target object. We, however, observed that such proposal methods provide unstable results on the plenoptic image sequences since the detectors often failed to find the target object even if we have used a reliable detector such as Faster R-CNN [32]. This is mainly because such detectors are not robust to blurs that the plenoptic images intrinsically have and are confused from the other objects.

The distance between the target object and camera is directly related to the size of the target object in the image plane. For instance, the size of the target object in the image becomes lager as the object approaches to the fixed camera. Since the focus index is tightly related to the distance between the object and camera, it is a strong cue to estimate the scale of the object in the image. In this section, we introduce a novel bounding box proposal method that takes both the optimal focus index and the motion of the object into account.

### 5.1. Scaling Factors

To derive appropriately sized proposals, we explore the focal stack parameters. Among various parameters we concentrate three variables, $\left(\mu_{min},\mu_{max}\right)$ and $\mu_{interval}$, related to the distance of the focal planes from the camera. Specifically, $\mu_{min}$ and $\mu_{max}$ indicate the range of the distances between focal planes and the camera, and $\mu_{interval}$ is a gap between two consecutive focal planes. Let us introduce a *scaling factor*$s^{t}$ at *t*th frame that determines the relative size change from the selected proposal in the previous frame t-1, based on difference in the focus indices ${\hat{k}}^{t-1}$ and ${\hat{k}}^{t}$ computed by our focus index selection algorithm described in Section 4.

**Figure 5:**
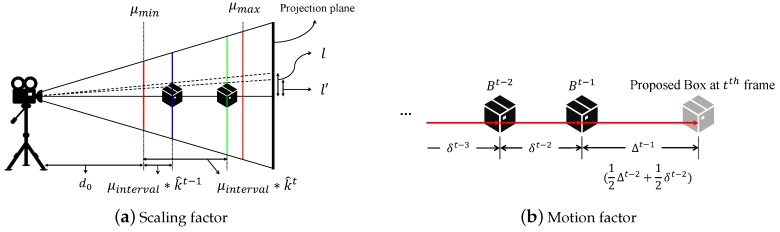
(**a**) This figure shows the geometric relationship between the object size in the image plane and the focus index. Since the focus index reflects the distance between the object and camera, the size of the object can be approximated based on the focus index changes. Our detailed derivation is given in Section 5.1. (**b**) This figure illustrates our motion factor formulation. Our underlying assumption is that the motion of object is not significantly changing from past a few frames. The displacement from the previous frame is then predicted as a linear combination of past displacements, where the weights are exponentially decreasing with respect to frame index distance.

Since size of the target object is proportionally decreasing to its distance from the camera, the relative scale between two frames *t* and t-1 can be expressed as follows:

(8) $$\begin{array}{c} \mathrm{size}\left(t\right)=\frac{d_{0}+\mu_{interval}\times {\hat{k}}^{t-1}}{d_{0}+\mu_{interval}\times {\hat{k}}^{t}}\times \mathrm{size}\left(t-1\right), \end{array}$$

where $d_{0}$ is the distance from camera to its closet focal plane. Figure 5a illustrates a geometric interpretation of the object size changes with respect to the focus index. However, it is hard to have the exact value of $d_{0}$ in practice, so we approximate $d_{0}$ as $\mu_{max}-\mu_{min}$. We then finally define our scaling factor $s^{t}$ as follows:

(9) $$\begin{array}{c} s^{t}=\frac{\left(\mu_{max}-\mu_{min}\right)+\mu_{interval}\times {\hat{k}}^{t-1}}{\left(\mu_{max}-\mu_{min}\right)+\mu_{interval}\times {\hat{k}}^{t}}. \end{array}$$

Intuitively, we produce smaller proposals when the focus index is increased from the previous frame and larger ones when the focus index is decreased. As a result, the width $\tilde{w}$ and heights $\tilde{h}$ of our generated proposals are shrank or enlarged as follows:

(10) $$\begin{array}{c} \tilde{w}={\hat{w}}^{t-1}+z_{s}\left(s^{t}-1\right){\hat{w}}^{t-1}\;\mathrm{and}\;\tilde{h}={\hat{h}}^{t-1}+z_{s}\left(s^{t}-1\right){\hat{h}}^{t-1}, \end{array}$$

where ${\hat{w}}^{t-1}$ and ${\hat{h}}^{t-1}$ are the width and height of the selected proposal at the previous frame t-1, respectively. The parameter $z_{s}$ is multiplied to generate proposals in multi-scale. When we propose a single scaled box, the $z_{s}$ should be 1. In evaluation (Section 6), we have proposed boxes with three different scales, $z_{s}=0,1$, and 2.

### 5.2. Motion Factors

In addition to predict the scale changes, estimating the location of the target object is also crucial to produce more accurate proposals. To estimate the location of the target object, we take the trajectory of target objects in the past frames into account. Specifically, we predict the 2D location $c^{t}={\left[{\tilde{x}}^{t},{\tilde{y}}^{t}\right]}^{T}$ at a frame *t* based on the center positions $c^{1}={\left[x^{1},y^{1}\right]}^{T},...,c^{t-1}={\left[x^{t-1},y^{t-1}\right]}^{T}$ of past tracking results $B^{1},B^{2},...,B^{t-1}$. Let us first define the displacement $\delta_{i}$ between *i* and i+1 frames as the follows:

(11) $$\begin{array}{c} \delta^{i}=c^{i+1}-c^{i}={\left[x^{i+1}-x^{i},y^{i+1}-y^{i}\right]}^{T} \end{array}$$

Our assumption on this task is that motion of the target object is not significantly changed from past few frames and highly likely to be similar to recent history of the trajectory. We design the following model by reflecting the assumption:

(12) $$\begin{array}{c} c^{t}=c^{t-1}+z_{m}\Delta^{t-1}, \end{array}$$

where

(13) $$\begin{array}{c} \Delta^{t-1}=\frac{1}{2}\delta^{t-2}+\frac{1}{4}\delta^{t-3}+\frac{1}{8}\delta^{t-4}+...+\frac{1}{2^{t-2}}\delta^{1}=\sum_{i=1}^{t-2}\frac{1}{2^{t-i-1}}\delta^{i}. \end{array}$$

and $z_{m}$ is a parameter for generating multiple bounding box proposals as we have in the scale factor (Section 5.1). The displacement of recent frames influences more on our prediction, and the factors are exponentially decreasing with respect to the distance of frame index. Since our estimated displacement is defined recursively, it can be efficiently computed as follows (Refer Figure 5b):

(14) $$\begin{array}{c} \Delta^{t-1}=\frac{1}{2}\Delta^{t-2}+\frac{1}{2}\delta^{t-2}. \end{array}$$

Once multiple bounding box proposals are generated based on various $z_{s}$ and $z_{m}$, we are required to select the best one among the candidates. We evaluate the proposals according to the image patch similarity. Specifically, the proposal which is the most similar to the tracking result at the previous frame $c(I_{{\hat{k}}_{t-1}}^{t-1},B^{t-1})$ is selected and feed to tracker. To compute the similarity among patches we employ the SSIM score [41].

## 6. Evaluation

In this section, we evaluate our proposed techniques for the object tracking on plenoptic image sequences.

### 6.1. Benchmarks

We first introduce the following benchmarks used in the experiments:

- **Seq #1**: The simplest one among three benchmarks. The target object is simply moving horizontally in the image plane and partially occluded. This sequence consists of 150 frames and the size of the focal stack is 14.
- **Seq #2**: The target object is moving far from the camera, so its size in the image is getting smaller. The occlusion is more serious than Seq #1. The number of frames and the size of the focal stack are 220 and 40, respectively.
- **Seq #3**: Contrary to Seq #2, the target object is approaching to the camera, so its size is getting larger. This sequence has 230 frames and the size of the focal stack is the same with Seq #2. The occlusion is the most serious among three sequences.

**Figure 6:**
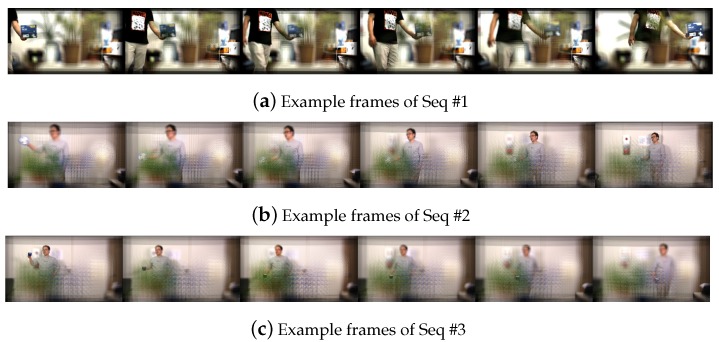
Several frames from our benchmark. Among images in the focal stack, we show the images selected by our proposed focus index selection method described in Section 4.

Please note that, the target objects of Seq #1, #2 and #3 are a paper box, an electric hand fan, and a cup, respectively. The cup in Seq #3 is a simple solid colored cup which does not have rich visual features. It is smaller than the other target objects while the Seq #3 has the most serious occlusions. These reasons lead the Seq #3 the most difficult scenario among tested benchmarks. Table 1 describes the parameters and Figure 6 shows several frames of benchmarks. We have used a plenoptic camera consisting of a 5×5 camera array to construct our benchmarks. Details about the equipment is described in [2,3].

### 6.2. Evaluation Protocol

All the tested methods are evaluated using two accuracy measures for the object tracking. For a sequence of estimated bounding boxes $B^{1},...,B^{N}$, a distance score is defined as the average of distances between centers of estimated and ground truth bounding boxes as follows:

(15) $$\begin{array}{c} \mathrm{dist}-\mathrm{score}\left(B^{1},...,B^{N}\right)=\frac{1}{N}\sum_{t=1}^{N}\sqrt{{\left(x^{t}-x_{GT}^{t}\right)}^{2}+{\left(y^{t}-y_{GT}^{t}\right)}^{2}}, \end{array}$$

where $\left(x^{t},y^{t}\right)$ and $\left(x_{GT}^{t},y_{GT}^{t}\right)$ are the center positions of estimated and ground truth boxes at frame index *t*, respectively. The second accuracy measure, an overlap score, is evaluating the intersection of the estimated and ground truth boxes. In the experiments we used a constant-sized bounding box for both estimation and ground truth, so the overlap score is defined as follows:

(16) $$\begin{array}{c} \mathrm{overlap}\;\mathrm{score}\left(B^{1},...,B^{N}\right)=\frac{1}{N}\sum_{t=1}^{N}\frac{|B^{t}\cap B_{GT}^{t}|}{|B^{t}\cup B_{GT}^{t}|}, \end{array}$$

where |·| is the number of pixels contained in the region. Please note that the ground truth bounding boxes are labeled by human. We conducted the experiment more than 10 times for each sequence with varying initial bounding boxes. Specifically, the number of trials on Seq #1, #2, and #3 are 18, 12, and 14 cases, respectively. The reported accuracy and timings are the averages of such many trials.

We mainly compare the following five methods:

- **Baseline #1** [2]: This method uses only the f-score to select a focus index and Boosting tracker [5].
- **Baseline #2** [3]: An extension from Baseline #1. This method additionally performs image sharpening process and generates proposals based on a fixed ratio of sizes. We set the ratio as 3% as suggested in [3].
- **Ours(B)**: Our method using fi-score (Section 4) to select a focus index, producing proposals based on the scaling and motion factors (Section 5), and using Boosting tracker [5].
- **Ours(M)**: Similar to Ours(B) but using MOSSE tracker [6].
- **Ours(MC)** Similar to Ours(M) but the MOSSE tracker is using CNN features [7].

All the tested methods are implemented in Python language, and all the experiments are conducted on a machine consisting of Intel i7-8700K CPU, 16 GB main memory, and NVIDIA GTX 1080 GPU.

In our focus index selection algorithm (Section 4), we employed image features to estimate visual similarity among image patches. We specifically used deep features extracted from the last fully connected layer of pre-trained version of VGG-16 [27] model which is publicly available. To train correlation filters of MOSSE tracker with CNN features, we used the activated feature map of the first convolution layer as suggested in [7]. Our proposal technique can produce multiple candidate boxes by applying different parameters $z_{s}$ and $z_{m}$. In experiment we mostly used 3 values, 0, 1, and 2, for each parameter, thus we considered 9 proposals in total.

### 6.3. Experimental Results

Table 2 shows the object-tracking accuracies for the tested method on three different benchmarks. Among techniques using the Boosting tracker, Baseline #1, #2, and Ours(B), Ours(B) provided the highest accuracy in all the tested datasets. Moreover, the second-best method, Baseline #2, is tightly coupled with the Boosting tracker. Its proposal algorithm is designed and optimized for the Boosting tracker, so Baseline #2 cannot accept other trackers such as MOSSE. On the other hand, our method can use any advanced trackers since there is not any assumption on the tracker.

By applying MOSSE tracker and its extension with CNN features to our technique, Ours(M) and Ours(MC), consistently outperformed the other tested methods including Ours(B). These experimental results verify that our method can provide even higher accuracy if we use more accurate 2D tracker.

To validate the benefits of our focus index selection technique (Section 4) we compared the tracking results based on two different focus index scoring schemes, f-score (Section 4.1) and fi-score (Section 4.3), and results of the experiments are given in Table 3. As reported in Table 3, our fi-score based on both the focus measure and visual similarity provides significantly higher tracking accuracy over the f-score used in Baseline #1 and #2. Please note that, we did not use any proposal algorithms in this experiment for a fair comparison.

**Figure 7:**
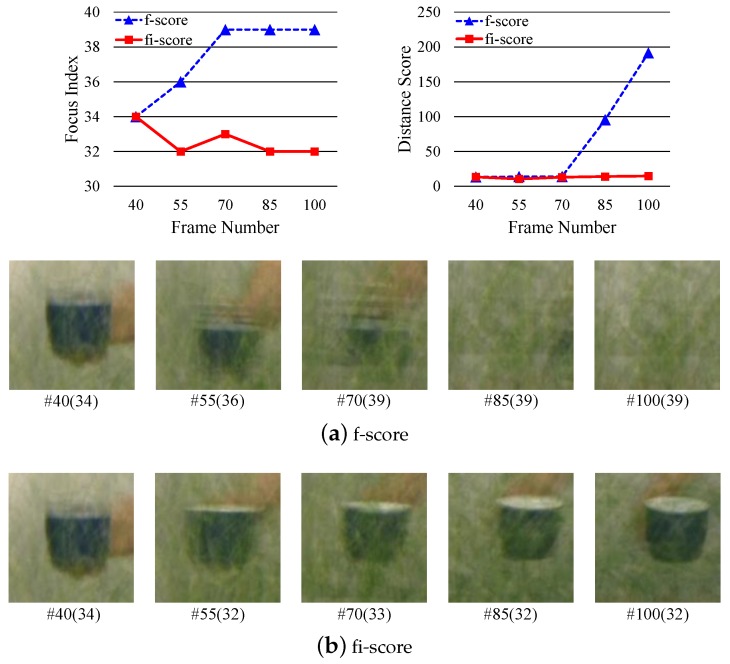
Qualitative comparison in Seq #3 between two scoring methods for the focus index selection, f-score and fi-score. A pair of values for each image patch denotes the frame index and the computed optimal focus index. Since the f-score only considers the focus measure, it can identify a wrong focus index when the target object gets behind the occluder. As shown in (a), the focus index selection based on f-score suggested an optimal focus index where the occluders are maximally focused. On the other hands, our fi-score-based focus index selection algorithm stably identifies appropriate focus indices over frames. Please note that, the quantitative results corresponding this figure is given in Table 3, and the proposal algorithms are excluded in this experiment.

**Figure 8:**
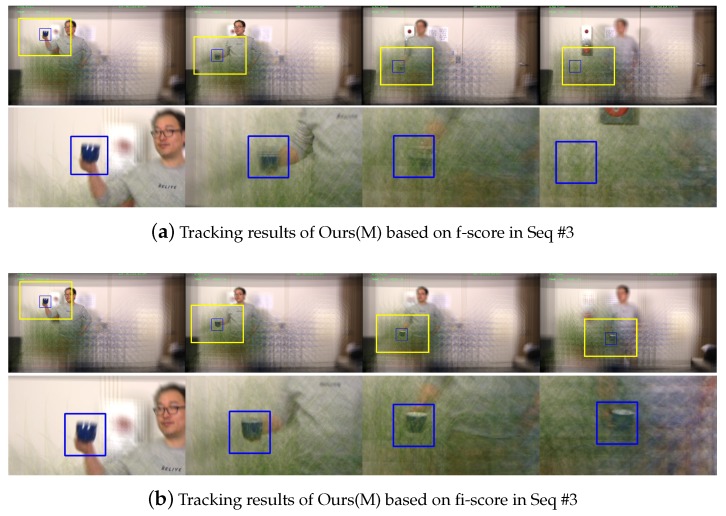
A comparison between the tracking results based on f-score and fi-score in Seq #3. The tracking process based on the f-score missed the target object once it was occluded because a wrong focus index is selected, while one with our fi-score was providing stable tracking performance. Please note that, we did not perform any candidate box proposal stage and MOSSE tracker is used in this experiment.

We also report the qualitative results corresponding to Table 3. Figure 7 shows the image patches at the position of the target object from the focus indices selected by f-score and fi-score. Since the f-score only relies on the focus measure, the selected focus index based on the f-score index can be wrong once the target object got behind an occluder. As shown in Figure 7a, the f-score-based technique identified the focus index where the occluder is maximally focused. On the other hands, our proposed fi-score robustly found appropriate focus indices over frames, since it considers not only the focus measure but also the visual consistency. The tracking results corresponding to this comparison is reported in Figure 8. The tracking process based on the f-score failed to locate the target object once it is occluded, while one with our fi-score was correctly estimating the target object. Please note that, in those experiments the proposal algorithms are excluded and MOSSE tracker was used.

We also conducted experiments of our method with and without proposals, and its results are shown in Table 4. As reported, our candidate box proposal algorithm consistently provided accuracy improvements. The improvement on Seq #2 was the largest among three sequences mainly because Seq #2 has more varied motions and object sizes compared to Seq #1, and #3. These results validate that our proposal algorithm based on scaling and motion factors assists trackers to accurately locate the target object.

**Figure 9:**
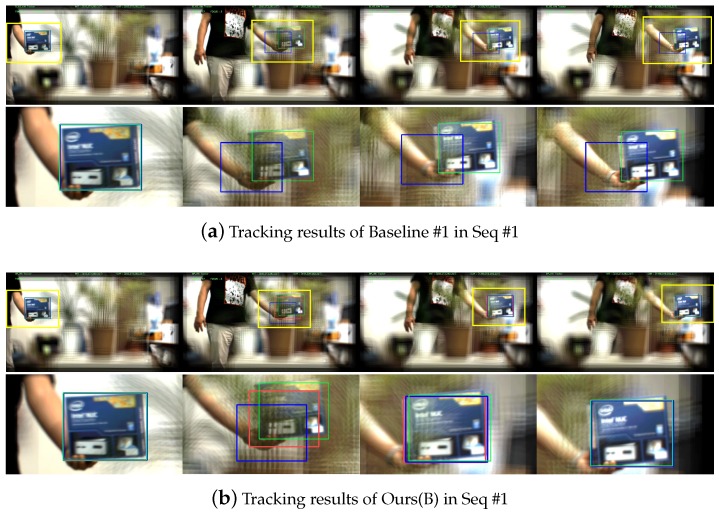
Qualitative analysis of the motion factor in our proposal algorithm. The yellow boxes in the first rows of (**a**,**b**) are magnified to the second rows for a clearer presentation. The green boxes are the ground truth bounding boxes while blue boxes are the tracking results. In the second columns, both methods lost the target object while it is moving fast. (**a**) Baseline #1 failed to recover its tracking capability in the following. (**b**) Ours(B), however, immediately relocated the target object by assistant of the proposal (red boxes). These qualitative results clearly validate the benefits of our proposal algorithm.

Let us report qualitative analysis of our proposal algorithm in Figure 9 by comparing Baseline #1 and Ours(B) in Seq #1. We mainly investigate our motion factor in the candidate box proposal stage. Figure 9a,b shows the tracking results of Baseline #1 and Ours(B), respectively. Baseline #1 failed to locate the target object when it is moving fast. Since most of trackers including Boosting-based techniques incrementally learn the appearance of the object, they hardly recover the tracking capability once they lost the object. On the other hand, our proposal algorithm considering the motion factor can assist the trackers to recover its tracking capability. Our tracker has lost the object between first and second columns in Figure 9b; however, it immediately relocated the target object based on the proposal bounding box as shown in the third columns. Please note that, the selected proposals are illustrated in red boxes.

**Figure 10:**
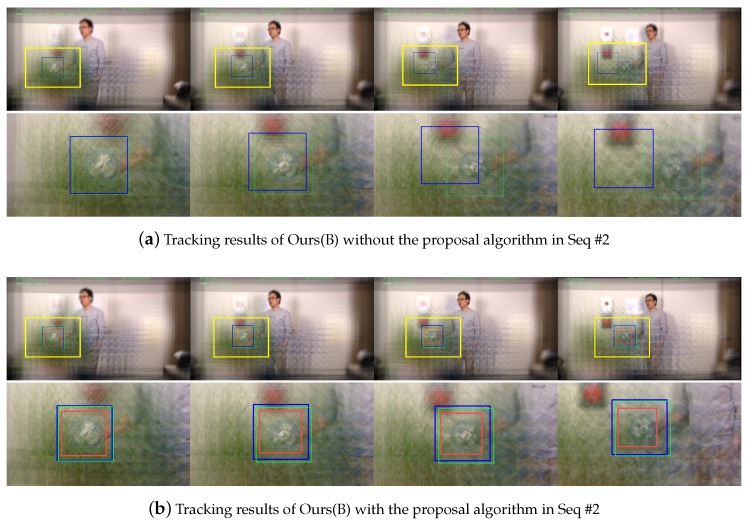
Qualitative evaluation of object tracking with/without the proposal algorithm in Seq #2. As the target object is getting smaller, our proposal algorithm generates the proposals which fit to the object. The same colors are used with Figure 9 for representing ground truth, tracking results, and proposals.

Another qualitative results to evaluate our proposal algorithm is given in Figure 10. Figure 10a,b show tracking results of Ours(B) without and with our proposal algorithm in Seq #2, respectively. When the size of the target object is getting smaller, as shown in the third column of the Figure 10a, Ours(B) without the proposals failed to estimate a correct location. On the other hand, our proposal algorithm generated suitable sized proposals (red boxes) and thus we could precisely locate the target object even with the scale changes.

Since our proposed focus index selection and candidate proposal techniques use image features to capture the content of the images, a choice of the feature extractor is fairly important. In all the aforementioned experiments, we used the VGG-16 network [27] as the feature extractor. Specifically, we used the last fully connected layer of VGG-16 in the focus index selection and the first convolution layer to train MC (MOSSE+CNN) tracker. The VGG-16 provides high accuracy in the image classification; however, it is known that its feature-extraction speed is quite slow. In order to see the performance of our proposed techniques when using lightweight feature extractor, we conducted experiments with SqueezeNet [40]. We also included the Inception-ResNet v2 [30] which showed one of the state-of-the-art classification performances recently. We report the tracking results with three different feature extractors, VGG-16, SqueezeNet and Inception-ResNet v2, in Table 5. The tracking accuracies are not significantly different. Since we have used pre-trained models publicly available for the feature extraction, the feature-extraction capability for particular objects can be diverse. Such diversity in the capability causes inconsistent performance tendency; however, our method still significantly outperformed the baseline techniques.

Let us finally report the average tracking speed. Baseline #1 and #2 took 0.1 and 0.16 seconds per frame, respectively. On the other hands, Ours(B), Ours(M), and Ours(MC) took 0.37, 0.32, and 0.70 s per frame on average when we used VGG-16 network as the feature extractor, respectively. As reported in Table 6, the tracking times of MOSSE tracker with VGG-16 and Inception-ResNet are similar. The MC tracker with the Inception-ResNet v2 provides the slowest tracking speed, because it has huge sized first convolution layer so that the longer time is consumed for updating correlation filters. On the other hand, our proposed techniques can be accelerated if we use computationally lighter models such as SqueezeNet. For instance, when we combined our proposed techniques with the MOSSE tracker and SqueezeNet as the feature extractor, our method provided both higher accuracy and faster speed compared to Baseline #2. Moreover, the fastest method among the tested combinations, our focus index selection method combined with SqueezeNet, provided significantly higher accuracy over Baseline #1 and #2.

## 7. Conclusions

We have presented a novel object-tracking method for plenoptic image sequences. To find an appropriate focal plane where the target object clearly appears, we have developed a content-aware focus index selection technique based on the visual consistency and focus measure. In addition to the focal plane selection, we have provided candidate box proposal scheme that considers two factors, a scale factor based on the change of the focus index and a motion factor estimated from the past trajectory of the target object, and selects an optimal proposal according to the content similarity. We have extensively evaluated our technique over the prior tracking methods for the plenoptic image sequences. Since our method is orthogonal to the choice of tracker for 2D images, we can take advantage of any advanced tracking algorithms designed for ordinary 2D image sequences. For the experiments, we have combined three popular trackers to our technique. According to the experimental results, our method has provided significantly higher accuracy over the compared methods. The performance improvements were consistent and significant. Those results clearly confirm the benefits of our proposed algorithms.

## Figures and Tables

**Table 1:** Parameters of the plenoptic image sequences used in the experiments.

	Seq #1	Seq #2	Seq #3
$\mu_{min}$	−0.9	−6.8	−6.7
$\mu_{max}$	−0.2	0.8	0.9
$\mu_{int}$	0.05	0.2	0.2
Focal Stack Size *M*	14	40	40
Frames	150	220	230

**Table 2:** Tracking accuracies (D and O indicate the distance and overlap scores, respectively.)

				Seq #1		Seq #2		Seq #3	
Method	Focus	Proposal	Tracker	D	O	D	O	D	O
Baseline #1 [2]	f-score	×	Boosting	44.502	0.852	59.997	0.712	142.796	0.576
Baseline #2 [3]	f-score	Fixed ratio	Boosting	39.293	0.867	55.916	0.735	41.101	0.822
Ours(B)	fi-score	Ours	Boosting	28.256	0.904	34.980	0.825	15.014	0.858
Ours(M)	fi-score	Ours	MOSSE	**8.400**	**0.959**	**26.584**	**0.882**	11.394	0.889
Ours(MC)	fi-score	Ours	MOSSE+CNN	11.978	0.946	27.469	0.879	**9.148**	**0.915**

**Table 3:** Comparison between f-score and fi-score with various trackers in Seq #3. Each pair of numbers denote the distance score and overlap score. Focus index selection based on our fi-score significantly outperform the f-score used in [2,3]. Please note that, the proposal algorithms are excluded for a fair comparison.

	Boosting	MOSSE	MOSSE+CNN
f-score	28.665/0.853	31.630/0.829	13.601/0.873
fi-score	19.984/0.842	11.534/0.888	9.807/0.909

**Table 4:** Accuracies of our methods with/without proposals.

	Seq #1		Seq #2		Seq #3	
	Distance	Overlap	Distance	Overlap	Distance	Overlap
Ours(M) w/o Proposal	8.789	0.957	43.868	0.807	11.534	0.888
Ours(M)	8.400	0.959	26.584	0.882	11.394	0.889
Ours(MC) w/o Proposal	14.523	0.936	43.683	0.810	9.807	0.909
Ours(MC)	11.978	0.946	27.469	0.879	9.148	0.915

**Table 5:** Average Distance scores with three different feature extractors, VGG-16, SqueezeNet and Inception-ResNet v2. P denotes our proposal algorithm.

	Seq #1			Seq #2			Seq #3		
	VGG	SQZ	IncRes	VGG	SQZ	IncRes	VGG	SQZ	IncRes
Ours(M) w/o P	8.789	8.624	8.627	43.868	4.957	15.967	11.534	11.509	23.347
Ours(M)	8.400	8.322	8.271	26.584	4.653	15.954	11.394	11.358	12.485
Ours(MC) w/o P	14.523	85.041	44.089	43.683	23.516	34.385	9.807	11.956	15.021
Ours(MC)	11.978	18.765	13.939	27.469	18.566	11.685	9.148	11.828	9.450

**Table 6:** Tracking times per frame with three different feature extractors, VGG-16, SqueezeNet and Inception-ResNet v2. The timings are measured with Seq #2 and #3.

	Time(s)/Frame		
	VGG	SQZ	IncRes
Ours(M) w/o Proposal	0.275	0.085	0.281
Ours(M)	0.319	0.155	0.322
Ours(MC) w/o Proposal	0.658	0.565	1.111
Ours(MC)	0.706	0.633	1.186
